# Effect of Polymorphisms of ABCB1 and MTHFR on Methotrexate-Related Toxicities in Adults With Hematological Malignancies

**DOI:** 10.3389/fonc.2021.759805

**Published:** 2021-12-24

**Authors:** Jian Han, Lu Liu, Li Meng, Huan Guo, Jin Zhang, Zhi-Qiang Han, Zhen-Ya Hong

**Affiliations:** ^1^ Department of Gastroenterology, Tongji Hospital of Tongji Medical College, Huazhong University of Science and Technology, Wuhan, China; ^2^ Department of Pharmacy, Tongji Hospital of Tongji Medical College, Huazhong University of Science and Technology, Wuhan, China; ^3^ Department of Hematology, Tongji Hospital of Tongji Medical College, Huazhong University of Science and Technology, Wuhan, China; ^4^ Department of Occupational and Environmental Health, School of Public Health, Tongji Medical College, Huazhong University of Science and Technology, Wuhan, China; ^5^ Department of Obstetrics and Gynecology, Tongji Hospital of Tongji Medical College, Huazhong University of Science and Technology, Wuhan, China

**Keywords:** high dose MTX, MTX-related toxicities, hematological malignancies, single nucleotide polymorphisms, ABCB1, MTHFR

## Abstract

Study of the association between single nucleotide polymorphisms (SNPs) of methotrexate (MTX) pathway genes and MTX-related toxicity in the treatment of hematological malignancies is popular. Here, we studied the association between SNPs of MTHFR and ABCB1 and MTX-related toxicity in 157 adult Chinese patients diagnosed with hematological malignancies. Patients were genotyped for MTHFR rs1801131, MTHFR rs1801133, and ABCB1 rs1045642 by fluorescence *in situ* hybridization. Patients with MTHFR rs1801133T allele had a significantly higher risk of hematopoietic toxicity compared with those with CC genotype (p=0.003). With respect to MTHFR rs1801131, patients with CC and AC genotypes had significantly lower frequency of hematopoietic toxicity than patients with AA genotype (p=0.044). In conclusion, we identified an important influence of the SNPs of ABCB1 and MTHFR on MTX-related hematopoietic toxicity in adults with hematological malignancies. To optimize high-dose (HD)-MTX therapy and reduce related hematopoietic toxicity, it is necessary to detect the SNPs of MTHFR and ABCB1 before initiating HD-MTX and deciding the optimal dose of MTX and duration of leucovorin rescue, according to genetic tests and disease type in adults with hematological malignancies.

## Introduction

The antimetabolic drug methotrexate (MTX) has been used to treat a variety of diseases, such as tumors and autoimmune diseases (1, 2). High-dose (HD)-MTX is a classic protocol for treating hematological malignancies (3). However, HD-MTX can lead to MTX-related toxicity, including mucositis (4), hepatotoxicity (5), nephrotoxicity (6) and hematopoietic toxicity (6). The pharmacokinetics of MTX and MTX-related toxicity differ among individuals, which can be partly explained by the single nucleotide polymorphisms (SNPs) of MTX pathway genes, including MTHFR and ABCB1 (7–14). A genome-wide association study (GWAS) was performed to identify germline polymorphisms for their association with MTX-induced neurotoxicity, and found that polymorphisms in genes related to neurogenesis may contribute to susceptibility to MTX-related neurotoxicity (15). However, there has been no GWAS about the relation between SNPs and other MTX-related toxicity, such as mucositis, hepatotoxicity, nephrotoxicity and myelosuppression. Methylene tetrahydrofolate reductase (MTHFR) is an important enzyme in folate metabolism, which is involved in nucleotide synthesis and DNA methylation (16, 17). ABC subfamily B member 1 (ABCB1), belongs to the ABC transporter subfamily, and MTX is pumped out from the cells by ABC subfamily transporters, including ABCB1 (7, 8). However, the results of previous research are discordant, which may be due to small sample sizes, different disease types and different chemotherapy regimens (8). Thus, our present research aimed to study the influence of SNPs of ABCB1 and MTHFR on MTX-related toxicity in adult Chinese patients diagnosed with hematological malignancies.

## Materials and Methods

### Patients

The study included 157 adult Chinese patients with acute lymphoblastic leukemia (ALL), chronic myeloid leukemia lymphoid blast crisis (CML-LBC), T-cell lymphoblastic lymphoma (TLBL), diffuse large B-cell lymphoma (DLBCL), NK/T cell lymphoma, mantle cell lymphoma (MCL), follicular lymphoma (FL), aggressive NK cell leukemia (ANKL) or Burkitt lymphoma. This study was attributed to Tongji Hospital affiliated to Huazhong University of Science and Technology, which is located in the city of Wuhan, Hubei Province, China. Patients were recruited from the Department of Hematology of Tongji Hospital between March 2017 and May 2019, and this was a retrospective biorepository study. The pathological types of non-Hodgkin’s lymphoma (NHL) were classified according to the WHO 2016 classification system (18).

### HD-MTX Treatment Protocols

The multiagent chemotherapeutic protocols used were SMILE (steroid, MTX, ifosfamide, L-asparaginase, etoposide) chemotherapy for patients diagnosed with NK/T cell lymphoma and ANKL, MA (MTX, Cytarabine/Ara-C) chemotherapy and hyper-CVAD (cytarabine, methotrexate) protocol-course B for patients diagnosed with TLBL, MCL, FL, DLBCL and Burkitt lymphoma, and MTX monotherapy, MA (MTX, Cytarabine/Ara-C) chemotherapy and MTX plus asparaginase for patients diagnosed with ALL and CML-LBC. All patients received high-dose MTX treatment, particularly 1g/m^2^~5g/m^2^ over 24h for patients diagnosed with ALL, CML-LBC, ANKL and TLBL, 1g/m^2^~4g/m^2^ over 24h for patients diagnosed with Burkitt lymphoma, MCL, DLBCL, FL and NK/T cell lymphoma, followed by leucovorin rescue. The infusion time between the different MTX treatments is the same for the different patient groups. MTX was administered as follows: 20% of the total dose was administered by intravenous infusion over 1 hour, and the remaining 80% over 23 hours. Leucovorin rescue was initiated with a 15 mg/m^2^ dose at 12h after the end of the HD-MTX infusion, and given every 6 hours, until the plasma MTX concentration was below 0.1μmol/L. A high plasma MTX level after 48h (> 0.1μmol/L) was defined as an indication for prolonged rescue. Monitoring of MTX concentration in plasma was carried out every day until the level was below 0.1μmol/L.

### Toxicity Evaluation

Toxicity was evaluated according to the National Cancer Institute Common Terminology Criteria for Adverse Events version 5.0 and included hematopoietic toxicity, as well as non-hematopoietic toxicity (hepatotoxicity, nephrotoxicity and mucositis). MTX-induced toxicity was assessed for the period between administration of MTX and the next course of chemotherapy. The subsequent course of chemotherapy was started during a period of 4 weeks after MTX infusion, following resolution of toxicity. Hepatotoxicity was determined by the presence of an increase in bilirubin and/or alanine transaminase and/or aspartate transaminase. Nephrotoxicity was determined by the presence of an increase in creatinine.

### Target Genes and Polymorphisms

Three SNPs including MTHFR rs1801133, MTHFR rs1801131 and ABCB1 rs1045642 were selected. **Table 1** shows the information of the target genes and polymorphisms.

**Table 1:** Genetic Polymorphisms.

SNPs(rs#)	Genes	Alleles(1/2)*	Location*	Function*	MAF
rs1801131	MTHFR	A/C	chr1:11854476	missense, Glu>Ala	15.71%
rs1801133	MTHFR	C/T	chr1:11856378	missense, Ala>Val	28.57%
rs1045642	ABCB1	T/C	chr7:87138645	coding synonym, Ile>Ile	30.48%

*Location and function according to NCBI GRCh38; 1: major allele; 2: minor allele.

### White Blood Cell (WBC) Extraction and SNP Genotyping

Ammonium chloride solution was used for extraction of WBCs from EDTA anticoagulant whole blood samples from the patients. Nucleic acid purification reagent (Sino-era JIYIN Tech CO.LTD, Beijing, China) was added. The polymorphisms within MTX pathway genes that include MTHFR rs1801133, MTHFR rs1801131 and ABCB1 rs1045642 were genotyped at the Tongji Hospital using the Fluotech48E fluorescence quantitative analyzer (Tianlong Science and Technology, Xi’an, China) by fluorescence *in situ* hybridization.

### Statistical Analysis

Toxicity was represented by the value 1 or 0, indicating whether an adverse event did or did not occur during MTX treatment. Binary logistic regression and multiple linear regression were used to examine the association of the MTX pathway gene polymorphisms with MTX-induced toxicity. We performed the association analysis adjusting for age and sex. All statistical results were considered significant at p<0.05. All statistical analyses were performed using SPSS version 25.0 (SPSS, IBM Corp., IL, USA).

## Results

### Patients, Distribution of Toxicity and Genotyping Characteristics

**Table 2** provides a summary of the characteristics of the patients, their clinical condition and the toxicity experienced. Most patients had leukemia (50.96%), followed by NHL (49.04%). All patients were analyzed together and there were differences in the number of patients developing each kind of toxicity. Hematopoietic toxicity was the most frequently observed, appearing in 91.08% of the patients, followed by mucositis (58.60%) and hepatotoxicity (31.85%). Nephrotoxicity was the least commonly observed toxicity appearing in 15.92% of the patients. We genotyped three SNPs in two genes (MTHFR and ABCB1). The selected SNPs (MTHFR rs1801131, MTHFR rs1801133, ABCB1 rs1045642) are shown in **Table 1**. All SNP markers studied were in Hardy–Weinberg equilibrium (HWE).

**Table 2:** Characteristics of patients, their clinical condition and toxicity experienced.

Gender	n (%)
Male	99 (63.06)
Female	58 (36.94)
**Disease**	**n (%)**
Leukemia	80 (50.96)
ALL and CML-LBC	75 (47.77)
ANKL	5 (3.19)
Non-Hodgkin’s lymphoma	77 (49.04)
TLBL	21 (13.38)
MCL	3 (1.91)
FL	1 (0.64)
DLBCL	26 (16.56)
Burkitt lymphoma	8 (5.09)
NK/T cell lymphoma	18 (11.46)
**Toxicity**	**n (%)**
Mucositis	92 (58.60)
Hepatotoxicity	50 (31.85)
Nephrotoxicity	25 (15.92)
Hematopoietic toxicity	143 (91.08)
**Disease**	**Chemotherapy**
NK/T cell lymphoma and ANKL	SMILE (steroid, MTX, ifosfamide, L-asparaginase, etoposide) MTX dose: 1g/m^2^~4g/m^2^ for NK/T cell lymphoma 1g/m^2^~5g/m^2^ for ANKL
TLBL, MCL, FL, DLBCL and Burkitt lymphoma	MA (MTX, Cytarabine/Ara-C) and hyper-CVAD (cytarabine, methotrexate)-course B MTX dose: 1g/m^2^~5g/m^2^ for TLBL 1g/m^2^~4g/m^2^ for Burkitt lymphoma, MCL, DLBCL, FL
ALL and CML-LBC	MTX monotherapy, MA (MTX, Cytarabine/Ara-C) and MTX plus asparaginase MTX dose: 1g/m^2^~5g/m^2^

### Pharmacogenetics of MTX

Mucositis, hepatotoxicity, nephrotoxicity and hematopoietic toxicity are common adverse effects attributed to the use of HD-MTX. To investigate genetic determinants for MTX-related toxicity after HD-MTX, information regarding toxicity during therapy was available for 157 patients. The relationship between SNPs within the MTX pathway genes and MTX-related toxicity among patients is shown in **Tables 3**–**13**.

**Table 3:** The relationship between MTHFR C677T, MTHFR A1298C, ABCB1 C3435T and MTX-related toxicities. (Total).

Polymorphisms (n)	Mucositis		Hepatotoxicity		Nephrotoxicity		Hematopoietic toxicity	
	P-value	OR (95% CI)	P-value	OR (95% CI)	P-value	OR (95% CI)	P-value	RC (95% CI)
**MTHFR C677T**								
CC (59)	–	–	–	–	–	–	–	–
CT+TT(98)	0.656	1.178 (0.572-2.425)	0.857	0.935 (0.449-1.947)	0.32	1.656 (0.613-4.471)	0.112	0.36 (-0.085-0.805)
**MTHFR A1298C**								
AA(103)	–	–	–	–	–	–	–	–
AC+CC(54)	0.432	1.341 (0.645-2.791)	0.609	1.216 (0.574-2.578)	0.411	1.522 (0.559-4.144)	0.946	-0.016 (-0.47-0.439)
**ABCB1 C3435T**								
CC(63)	–	–	–	–	–	–	–	–
CT+TT(94)	0.432	1.32 (0.661-2.637)	0.687	1.16 (0.565-2.382)	0.179	0.517 (0.198-1.353)	0.301	-0.227 (-0.658-0.205)
**Gender**								
Female(58)	–	–	–	–	–	–	–	–
Male(99)	0.264	1.487 (0.741-2.983)	0.131	0.584 (0.29-1.174)	0.141	2.181 (0.773-6.155)	0.4	-0.184 (-0.615-0.247)
**Age**	0.25	0.986 (0.962-1.01)	0.351	0.988 (0.963-1.013)	0.262	1.02 (0.986-1.055)	0.041	-0.015 (-0.029–0.001)

**Table 4:** The relationship between MTHFR C677T, MTHFR A1298C, ABCB1 C3435T and MTX-related toxicities.

Polymorphisms (n)	Mucositis		Hepatotoxicity		Nephrotoxicity		Hematopoietic toxicity	
	P-value	OR (95% CI)	P-value	OR (95% CI)	P-value	OR (95% CI)	P-value	RC (95% CI)
**MTHFR C677T**								
CC (38)	–	–	–	–	–	–	–	–
CT+TT (59)	0.788	0.886 (0.367-2.141)	0.912	1.055 (0.409-2.717)	0.462	1.559 (0.478-5.082)	0.051	0.52 (-0.003-1.043)
**MTHFR A1298C**								
AA (60)	–	–	–	–	–	–	–	–
AC+CC (37)	0.354	1.53 (0.622-3.762)	0.58	1.306 (0.508-3.36)	0.224	2.072 (0.64-6.706)	0.916	0.028 (-0.501-0.557)
**ABCB1 C3435T**								
CC (45)	–	–	–	–	–	–	–	–
CT+TT (52)	0.954	0.976 (0.418-2.276)	0.779	1.138 (0.459-2.822)	0.146	0.427 (0.135-1.346)	0.171	-0.349 (-0.852-0.153)
**Gender**								
Female (34)	–	–	–	–	–	–	–	–
Male (63)	0.151	1.892 (0.792-4.521)	0.315	0.623 (0.248-1.566)	0.092	3.268 (0.826-12.94)	0.762	0.08 (-0.443-0.603)
**Age**	0.434	0.987 (0.956-1.019)	0.485	0.988 (0.954-1.023)	0.58	1.012 (0.971-1.054)	0.738	-0.003 (-0.022-0.016)

(TLBL & ALL & CML-LBC).

**Table 5:** The relationship between MTHFR C677T, MTHFR A1298C, ABCB1 C3435T and MTX-related toxicities.

Polymorphisms (n)	Mucositis		Hepatotoxicity		Nephrotoxicity		Hematopoietic toxicity	
	P-value	OR (95% CI)	P-value	OR (95% CI)	P-value	OR (95% CI)	P-value	RC (95% CI)
**MTHFR C677T**								
CC (14)	–	–	–	–	–	–	–	–
CT+TT (24)	0.064	5.511 (0.905-33.55)	0.833	0.84 (0.167-4.236)	0.999	0 (0-)	0.056	1.038 (-0.03-2.106)
**MTHFR A1298C**								
AA (26)	–	–	–	–	–	–	–	–
AC+CC (12)	0.803	1.256 (0.209-7.561)	0.281	2.831 (0.427-18.787)	0.999	6.522*10^12^ (0-)	0.221	0.721 (-0.455-1.896)
**ABCB1 C3435T**								
CC (13)	–	–	–	–	–	–	–	–
CT+TT (25)	0.085	4.644 (0.807-26.719)	0.422	2.044 (0.357-11.693)	0.997	6.378*10^24^ (0-)	0.431	0.411 (-0.64-1.462)
**Gender**								
Female (15)	–	–	–	–	–	–	–	–
Male (23)	0.526	0.581 (0.109-3.107)	0.762	0.762 (0.131-4.421)	0.997	0 (0-)	0.084	-0.96 (-2.059-0.138)
**Age**	0.996	1 (0.946-1.057)	0.365	0.972 (0.915-1.033)	0.991	421.686 (0-)	0.172	-0.026 (-0.063-0.012)

(DLBCL & Burkitt lymphoma & MCL & FL).

**Table 6:** The relationship between MTHFR C677T, MTHFR A1298C, ABCB1 C3435T and MTX-related toxicities.

Polymorphisms (n)	Mucositis		Hepatotoxicity		Nephrotoxicity		Hematopoietic toxicity	
	P-value	OR (95% CI)	P-value	OR (95% CI)	P-value	OR (95% CI)	P-value	RC (95% CI)
**MTHFR C677T**								
CC (7)	–	–	–	–	–	–	–	–
CT+TT (15)	0.999	0 (0-)	0.841	1.319 (0.088-19.834)	0.819	1.31 (0.129-13.277)	0.154	-1.006 (-2.435-0.424)
**MTHFR A1298C**								
AA (17)	–	–	–	–	–	–	–	–
AC+CC (5)	0.998	7.669*10^22^ (0-)	0.08	0.046 (0.001-1.454)	0.968	1.074 (0.034-33.44)	0.309	-0.971 (-2.935-0.993)
**ABCB1 C3435T**								
CC (5)	–	–	–	–	–	–	–	–
CT+TT (17)	0.998	0 (0-)	0.195	0.09 (0.002-3.441)	0.301	0.249 (0.018-3.472)	0.748	-0.254 (-1.908-1.4)
**Gender**								
Female (9)	–	–	–	–	–	–	–	–
Male (13)	0.998	0 (0-)	0.078	0.093 (0.007-1.3)	0.701	1.538 (0.171-13.849)	0.749	-0.205 (-1.544-1.133)
**Age**	0.421	0.94 (0.809-1.092)	0.299	0.937 (0.828-1.06)	0.683	0.979 (0.885-1.083)	0.667	-0.012 (-0.071-0.047)

(NK/T cell lymphoma & ANKL).

**Table 7:** The relationship between MTHFR C677T, MTHFR A1298C, ABCB1 C3435T and MTX-related toxicities.

Polymorphisms (n)	Mucositis		Hepatotoxicity		Nephrotoxicity		Hematopoietic toxicity	
	P-value	OR (95% CI)	P-value	OR (95% CI)	P-value	OR (95% CI)	P-value	RC (95% CI)
**MTHFR C677T**								
CC (32)	–	–	–	–	–	–	–	–
CT+TT (67)	0.68	1.212 (0.486-3.019)	0.462	1.472 (0.526-4.123)	0.276	2.741 (0.448-16.784)	0.311	0.29 (-0.275-0.854)
**MTHFR A1298C**								
AA (59)	–	–	–	–	–	–	–	–
AC+CC (40)	0.638	1.232 (0.516-2.941)	0.359	1.559 (0.603-4.032)	0.387	1.977 (0.422-9.25)	0.96	-0.013 (-0.549-0.523)
**ABCB1 C3435T**								
CC (42)	–	–	–	–	–	–	–	–
CT+TT (57)	0.501	1.34 (0.572-3.141)	0.456	1.435 (0.556-3.705)	0.438	0.515 (0.096-2.756)	0.753	-0.084 (-0.612-0.444)
**Gender**								
Female (36)	–	–	–	–	–	–	–	–
Male (63)	0.79	1.122 (0.479-2.628)	0.417	0.683 (0.273-1.713)	0.121	5.866 (0.628-54.781)	0.316	-0.267 (-0.793-0.259)
**Age**	0.439	0.988 (0.959-1.018)	0.778	0.995 (0.963-1.029)	0.245	1.032 (0.978-1.089)	0.112	-0.015 (-0.034-0.004)

(Dose: 1-2g/m2).

**Table 8:** The relationship between MTHFR C677T, MTHFR A1298C, ABCB1 C3435T and MTX-related toxicities.

Polymorphisms (n)	Mucositis		Hepatotoxicity		Nephrotoxicity		Hematopoietic toxicity	
	P-value	OR (95% CI)	P-value	OR (95% CI)	P-value	OR (95% CI)	P-value	RC (95% CI)
**MTHFR C677T**								
CC (24)	–	–	–	–	–	–	–	–
CT+TT (43)	0.684	0.799 (0.271-2.355)	0.928	1.056 (0.323-3.456)	0.297	2.632 (0.427-16.212)	0.804	0.082 (-0.578-0.743)
**MTHFR A1298C**								
AA (41)	–	–	–	–	–	–	–	–
AC+CC (26)	0.726	1.209 (0.418-3.496)	0.393	1.651 (0.523-5.21)	0.594	1.566 (0.301-8.146)	0.48	-0.231 (-0.881-0.419)
**ABCB1 C3435T**								
CC (29)	–	–	–	–	–	–	–	–
CT+TT (38)	0.822	0.886 (0.31-2.536)	0.893	0.924 (0.294-2.905)	0.593	0.625 (0.112-3.499)	0.337	-0.311 (-0.954-0.331)
**Gender**								
Female (23)	–	–	–	–	–	–	–	–
Male (44)	0.909	1.063 (0.371-3.048)	0.421	0.629 (0.203-1.946)	0.13	5.715 (0.6-54.415)	0.763	-0.098 (-0.745-0.549)
**Age**	0.668	0.992 (0.956-1.029)	0.721	0.993 (0.953-1.033)	0.181	1.039 (0.982-1.099)	0.564	-0.007 (-0.029-0.016)

(TLBL & ALL & CML-LBC Dose: 1-2g/m^2^).

**Table 9:** The relationship between MTHFR C677T, MTHFR A1298C, ABCB1 C3435T and MTX-related toxicities.

Polymorphisms (n)	Mucositis		Hepatotoxicity		Nephrotoxicity		Hematopoietic toxicity	
	P-value	OR (95% CI)	P-value	OR (95% CI)	P-value	OR (95% CI)	P-value	RC (95% CI)
**MTHFR C677T**								
CC (7)	–	–	–	–	–	–	–	–
CT+TT (18)	0.128	7.468 (0.56-99.641)	0.424	2.945 (0.209-41.601)	–	(-)	0.17	0.885 (-0.414-2.183)
**MTHFR A1298C**								
AA (15)	–	–	–	–	–	–	–	–
AC+CC (10)	0.973	0.959 (0.086-10.738)	0.478	2.676 (0.176-40.658)	–	(-)	0.309	0.72 (-0.722-2.162)
**ABCB1 C3435T**								
CC (10)	–	–	–	–	–	–	–	–
CT+TT (15)	0.143	5.888 (0.55-62.986)	0.23	5.535 (0.339-90.336)	–	(-)	0.166	0.889 (-0.402-2.179)
**Gender**								
Female (10)	–	–	–	–	–	–	–	–
Male (15)	0.698	0.617 (0.054-7.099)	0.906	0.848 (0.056-12.913)	–	(-)	0.123	-1.098 (-2.524-0.328)
**Age**	0.992	1 (0.93-1.075)	0.495	0.97 (0.889-1.059)	–	(-)	0.5	-0.015 (-0.062-0.031)

(DLBCL & Burkitt lymphoma & MCL & FL Dose: 1-2g/m^2^).

**Table 10:** The relationship between MTHFR C677T, MTHFR A1298C, ABCB1 C3435T and MTX-related toxicities.

Polymorphisms (n)	Mucositis		Hepatotoxicity		Nephrotoxicity		Hematopoietic toxicity	
	P-value	OR (95% CI)	P-value	OR (95% CI)	P-value	OR (95% CI)	P-value	RC (95% CI)
**MTHFR C677T**								
CC (27)	–	–	–	–	–	–	–	–
CT+TT (31)	0.88	1.102 (0.312-3.896)	0.412	0.617 (0.195-1.955)	0.592	1.397 (0.411-4.754)	0.19	0.511 (-0.261-1.283)
**MTHFR A1298C**								
AA (44)	–	–	–	–	–	–	–	–
AC+CC (14)	0.236	2.54 (0.544-11.847)	0.908	0.918 (0.215-3.913)	0.603	1.49 (0.332-6.691)	0.68	0.2 (-0.767-1.166)
**ABCB1 C3435T**								
CC (21)	–	–	–	–	–	–	–	–
CT+TT (37)	0.918	0.926 (0.214-4.011)	0.945	1.05 (0.262-4.202)	0.641	0.708 (0.166-3.014)	0.097	-0.773 (-1.691-0.146)
**Gender**								
Female (22)	–	–	–	–	–	–	–	–
Male (36)	0.092	3.101 (0.83-11.588)	0.196	0.461 (0.142-1.49)	0.592	1.422 (0.393-5.147)	0.974	0.013 (-0.791-0.817)
**Age**	0.712	0.99 (0.937-1.045)	0.183	0.965 (0.916-1.017)	0.921	0.997 (0.945-1.053)	0.615	-0.007 (-0.034-0.02)

(Dose: 2.5-5g/m^2^).

**Table 11:** The relationship between MTHFR C677T, MTHFR A1298C, ABCB1 C3435T and MTX-related toxicities.

Polymorphisms (n)	Mucositis		Hepatotoxicity		Nephrotoxicity		Hematopoietic toxicity	
	P-value	OR (95% CI)	P-value	OR (95% CI)	P-value	OR (95% CI)	P-value	RC (95% CI)
**MTHFR C677T**								
CC (14)	–	–	–	–	–	–	–	–
CT+TT (16)	0.586	0.592 (0.09-3.907)	0.997	1.003 (0.187-5.387)	0.791	0.786 (0.132-4.664)	***0.003***	**1.388 (0.506-2.269)**
**MTHFR A1298C**								
AA (19)	–	–	–	–	–	–	–	–
AC+CC (11)	0.203	3.717 (0.492-28.064)	0.798	0.79 (0.13-4.813)	0.206	3.434 (0.508-23.228)	0.361	0.411 (-0.5-1.322)
**ABCB1 C3435T**								
CC (16)	–	–	–	–	–	–	–	–
CT+TT (14)	0.752	1.367 (0.196-9.51)	0.264	2.898 (0.447-18.775)	0.857	0.838 (0.123-5.709)	0.055	-0.913 (-1.845-0.019)
**Gender**								
Female (11)	–	–	–	–	–	–	–	–
Male (19)	0.024	9.006 (1.327-61.116)	0.404	0.475 (0.083-2.725)	0.349	2.623 (0.349-19.704)	0.402	0.373 (-0.53-1.276)
**Age**	0.268	0.955 (0.88-1.036)	0.244	0.949 (0.87-1.036)	0.219	0.938 (0.848-1.038)	0.257	0.023 (-0.018-0.064)

(TLBL & ALL & CML-LBC Dose: 2.5-5g/m^2^). Bold values means that p value ＜0.05, and the related data is significant statistically.

**Table 12:** The relationship between MTHFR C677T, MTHFR A1298C, ABCB1 C3435T and MTX-related toxicities.

Polymorphisms (n)	Mucositis		Hepatotoxicity		Nephrotoxicity		Hematopoietic toxicity	
	P-value	OR (95% CI)	P-value	OR (95% CI)	P-value	OR (95% CI)	P-value	RC (95% CI)
**MTHFR C677T**								
CC (7)	–	–	–	–	–	–	–	–
CT+TT (6)	0.544	2.799 (0.101-77.641)	0.997	0 (0-)	0.998	0 (0-)	0.423	1.17 (-2.079-4.42)
**MTHFR A1298C**								
AA (11)	–	–	–	–	–	–	–	–
AC+CC (2)	0.469	3.918 (0.097-157.769)	0.998	7.99*10^31^ (0-)	0.999	2.74*10^12^ (0-)	0.27	1.86 (-1.808-5.529)
**ABCB1 C3435T**								
CC (3)	–	–	–	–	–	–	–	–
CT+TT (10)	0.997	0.993 (0.018-55.127)	1	0 (0-)	1	0.001 (0-)	0.552	-1.034 (-4.95-2.881)
**Gender**								
Female (5)	–	–	–	–	–	–	–	–
Male (8)	0.809	0.721 (0.051-10.227)	1	0 (0-)	0.998	0 (0-)	0.622	-0.582 (-3.249-2.086)
**Age**	0.635	1.033 (0.902-1.184)	0.997	158.601 (0-)	0.996	835.574 (0-)	0.74	-0.019 (-0.151-0.113)

(DLBCL & Burkitt lymphoma & MCL & FL Dose: 2.5-5g/m^2^).

**Table 13:** The relationship between MTHFR C677T, MTHFR A1298C, ABCB1 C3435T and MTX-related toxicities.

Polymorphisms (n)	Mucositis		Hepatotoxicity		Nephrotoxicity		Hematopoietic toxicity	
	P-value	OR (95% CI)	P-value	OR (95% CI)	P-value	OR (95% CI)	P-value	RC (95% CI)
**MTHFR C677T**								
CC (6)	–	–	–	–	–	–	–	–
CT+TT (9)	0.999	0 (0-)	0.953	1.112 (0.032-39.075)	0.787	1.387 (0.13-14.801)	0.279	-0.864 (-2.559-0.831)
**MTHFR A1298C**								
AA (14)	–	–	–	–	–	–	–	–
AC+CC (1)	1	3.936 (0-)	1	0 (0-)	1	0 (0-)	***0.044***	**-3.62 (-7.123—0.116)**
**ABCB1 C3435T**								
CC (2)	–	–	–	–	–	–	–	–
CT+TT (13)	0.999	0 (0-)	0.999	0 (0-)	0.714	0.561 (0.026-12.332)	0.75	-0.347 (-2.738-2.045)
**Gender**								
Female (6)	–	–	–	–	–	–	–	–
Male (9)	0.999	0 (0-)	0.153	0.061 (0.001-2.832)	0.904	0.866 (0.084-8.925)	0.354	-0.757 (-2.51-0.995)
**Age**	0.701	1.052 (0.811-1.365)	0.215	0.881 (0.721-1.076)	0.716	0.981 (0.883-1.089)	0.864	-0.006 (-0.077-0.066)

(NK/T cell lymphoma & ANKL Dose: 2.5-5g/m^2^). Bold values means that p value < 0.05, and the related data is significant statistically.

SNPs and MTX-Related Mucositis

With regard to all patients, patients with TLBL, ALL and CML-LBC, patients with DLBCL, Burkitt lymphoma, MCL and FL, and patients with NK/T cell lymphoma and ANKL, no polymorphism was found to be significantly associated with mucositis (**Tables 3**–**6**). When the dose of MTX was 1-2g/m^2^, with regard to all patients, patients with TLBL, ALL and CML-LBC, and patients with DLBCL, Burkitt lymphoma, MCL and FL, no polymorphism was found to be significantly associated with mucositis (**Tables 7**–**9**). When the dose of MTX was 2.5-5g/m^2^, with regard to all patients, patients with TLBL, ALL and CML-LBC, patients with DLBCL, Burkitt lymphoma, MCL and FL, and patients with NK/T cell lymphoma and ANKL, no polymorphism was found to be significantly associated with mucositis (**Tables 10**–**13**).

#### SNPs and MTX-Related Hepatotoxicity

With regard to all patients, patients with TLBL, ALL and CML-LBC, patients with DLBCL, Burkitt lymphoma, MCL and FL, and patients with NK/T cell lymphoma and ANKL, no polymorphism was significantly associated with hepatotoxicity (**Tables 3**–**6**). When the dose of MTX was 1-2g/m^2^, with regard to all patients, patients with TLBL, ALL and CML-LBC, and patients with DLBCL, Burkitt lymphoma, MCL and FL, no polymorphism was significantly associated with hepatotoxicity (**Tables 7**–**9**). When the dose of MTX was 2.5-5g/m^2^, with regard to all patients, patients with TLBL, ALL and CML-LBC, patients with DLBCL, Burkitt lymphoma, MCL and FL, and patients with NK/T cell lymphoma and ANKL, no polymorphism was significantly associated with hepatotoxicity (**Tables 10**–**13**).

#### SNPs and MTX-Related Nephrotoxicity

With regard to all patients, patients with TLBL, ALL and CML-LBC, patients with DLBCL, Burkitt lymphoma, MCL and FL, and patients with NK/T cell lymphoma and ANKL, no polymorphism was significantly associated with nephrotoxicity (**Tables 3**–**6**). When the dose of MTX was 1-2g/m^2^, with regard to all patients, patients with TLBL, ALL and CML-LBC, and patients with DLBCL, Burkitt lymphoma, MCL and FL, no polymorphism was significantly associated with nephrotoxicity (**Tables 7**–**9**). When the dose of MTX was 2.5-5g/m^2^, with regard to all patients, patients with TLBL, ALL and CML-LBC, patients with DLBCL, Burkitt lymphoma, MCL and FL, and patients with NK/T cell lymphoma and ANKL, no polymorphism was significantly associated with nephrotoxicity (**Tables 10**–**13**).

#### SNPs and MTX-Related Hematopoietic Toxicity

With regard to all patients, no polymorphism was significantly associated with hematopoietic toxicity (**Table 3**). In patients with TLBL, ALL and CML-LBC, no polymorphism was significantly associated with hematopoietic toxicity (**Table 4**). However, hematopoietic toxicity occurred more frequently in patients with the MTHFR rs1801133T allele (TT and CT) than in those with the CC genotype (p=0.051) (**Table 4**). In patients with DLBCL, Burkitt lymphoma, MCL and FL, no polymorphism was significantly associated with hematopoietic toxicity (**Table 5**). However, hematopoietic toxicity occurred more frequently in patients with the MTHFR rs1801133T allele (TT and CT) than in those with the CC genotype (p=0.056) (**Table 5**). In patients with NK/T cell lymphoma and ANKL, no polymorphism was significantly associated with hematopoietic toxicity (**Table 6**). When the dose of MTX was 1-2g/m^2^, no polymorphism was significantly associated with hematopoietic toxicity (**Table 7**). In patients with TLBL, ALL and CML-LBC when the dose of MTX was 1-2g/m^2^, no polymorphism was significantly associated with hematopoietic toxicity (**Table 8**). In patients with DLBCL, Burkitt lymphoma, MCL and FL when the dose of MTX was 1-2g/m^2^, no polymorphism was significantly associated with hematopoietic toxicity (**Table 9**). When the dose of MTX was 2.5-5g/m^2^, no polymorphism was significantly associated with hematopoietic toxicity (**Table 10**). In patients with TLBL, ALL and CML-LBC when the dose of MTX was 2.5-5g/m^2^, with regard to MTHFR rs1801133, patients with the CT and TT genotype had a significantly higher risk of developing hematopoietic toxicity than those with the CC genotype (p=0.003) (**Table 11**). In patients with TLBL, ALL and CML-LBC when the dose of MTX was 2.5-5g/m^2^, hematopoietic toxicity occurred less frequently in patients with the ABCB1 rs1045642T allele (TT and CT) than in those with the CC genotype (p=0.055) (**Table 11**). In patients with DLBCL, Burkitt lymphoma, MCL and FL when the dose of MTX was 2.5-5g/m^2^, no polymorphism was significantly associated with hematopoietic toxicity (**Table 12**). In patients with NK/T cell lymphoma and ANKL when the dose of MTX was 2.5-5g/m^2^, with regard to MTHFR rs1801131, patients with the CC and AC genotype had a significantly lower risk of developing hematopoietic toxicity than those with the AA genotype (p=0.044) (**Table 13**).

## Discussion

Study of the association between SNPs of MTX pathway genes and MTX-related toxicity has attracted much attention. Here, we studied the relation between SNPs of MTHFR and ABCB1 and MTX-related toxicity in 157 adult Chinese patients diagnosed with hematological malignancies. We found that ABCB1 rs1045642, MTHFR rs1801133 and MTHFR rs1801131 were all associated with MTX-related hematopoietic toxicity, but none of the three SNPs was associated with MTX-related mucositis, hepatotoxicity or nephrotoxicity. The conclusions of previous studies in this field are inconsistent, which may have resulted from small sample sizes, different disease types and different chemotherapy regimens (8). In our study, we found that no polymorphism was significantly associated with mucositis, hepatotoxicity, nephrotoxicity or hematopoietic toxicity in the total population. This might have been because the dose of MTX ranged from 1 to 5 g/m^2^ and that we included a wide variety of disease types. To exclude the influence of the confounding factors such as dose of MTX and type of hematological malignancies, the unique feature of our study is that we divided the patients into subgroups according to disease type and dose of MTX. According to the type of hematological malignancies, we divide the subjects into 3 subgroups. The first subgroup includes TLBL, ALL and CML-LBC. The second subgroup corresponds to B cell lymphomas which include DLBCL, Burkitt lymphoma, MCL and FL. The third subgroup includes NK/T cell lymphoma and ANKL. According to the dose of MTX, we divide the subjects into 2 subgroups, namely high-dose group and low-dose group. The dose of MTX corresponding to low-dose group is 1-2 g/m^2^, and the dose of MTX corresponding to high-dose group is 2.5-5 g/m^2^.

In patients with TLBL, ALL and CML-LBC, no polymorphism was found to be significantly associated with mucositis, hepatotoxicity or nephrotoxicity. However, hematopoietic toxicity occurred more frequently in patients with the MTHFR rs1801133T allele (TT and CT) than in those with the CC genotype (p=0.051) (**Table 4**). This subgroup contained only 97 patients, including 38 with MTHFR rs1801133 CC genotype and 59 with MTHFR rs1801133 CT and TT genotypes. We thought that if we enlarged the sample size or divided this subgroup into high-dose and low-dose groups and conducted statistical analysis, a significant difference would appear. However, in patients with TLBL, ALL and CML-LBC when the dose of MTX was 1-2 g/m^2^, no polymorphism was significantly associated with hematopoietic toxicity (**Table 8**). In patients with TLBL, ALL and CML-LBC when the dose of MTX was 2.5-5 g/m^2^, with regard to MTHFR rs1801133, patients with the CT and TT genotypes had a significantly higher risk of hematopoietic toxicity than patients with the CC genotype had (p=0.003) (**Table 11**). Therefore, if the dose of MTX is lower than 2 g/m^2^, in patients with TLBL, ALL and CML-LBC, it is not necessary to detect the gene polymorphisms, adjust the dose of MTX or lengthen the duration of leucovorin rescue. Conversely, if the dose of MTX is higher than 2.5 g/m^2^, in patients with TLBL, ALL and CML-LBC, it is necessary to detect the genotype of MTHFR rs1801133 and adjust the dose of MTX or lengthen the duration of leucovorin rescue in patients with the CT and TT genotypes in order to decrease the risk of developing hematopoietic toxicity.

In patients with DLBCL, Burkitt lymphoma, MCL and FL, no polymorphism was significantly associated with mucositis, hepatotoxicity or nephrotoxicity. However, hematopoietic toxicity occurred more frequently in patients with the MTHFR rs1801133T allele (TT and CT) than in patients with the CC genotype (p=0.056) (**Table 5**). This subgroup contained only 38 patients, including 14 with MTHFR rs1801133 CC genotype and 24 with MTHFR rs1801133 CT and TT genotypes. We thought that if we enlarged the sample size or divided this subgroup into high-dose and low-dose groups and conducted statistical analysis, a significant difference would appear. However, in patients with DLBCL, Burkitt lymphoma, MCL and FL when the dose of MTX was 1-2 g/m^2^, no polymorphism was significantly associated with mucositis, hepatotoxicity, nephrotoxicity or hematopoietic toxicity. Similarly, in patients with DLBCL, Burkitt lymphoma, MCL and FL when the dose of MTX was 2.5-5 g/m^2^, no polymorphism was significantly associated with mucositis, hepatotoxicity, nephrotoxicity or hematopoietic toxicity. Therefore, in future research, we should enlarge the number of patients with DLBCL, Burkitt lymphoma, MCL and FL, in order to draw a definite conclusion.

In patients with NK/T cell lymphoma and ANKL, no polymorphism was significantly associated with mucositis, hepatotoxicity, nephrotoxicity or hematopoietic toxicity. This subgroup contained only 22 patients. We thought that if we enlarged the sample size or divided this subgroup into high-dose and low-dose groups and conducted statistical analysis, a significant difference would appear. In patients with NK/T cell lymphoma and ANKL when the dose of MTX was 2.5-5 g/m^2^, with regard to MTHFR rs1801131, patients with the CC and AC genotypes had a significantly lower risk of developing hematopoietic toxicity than patients with the AA genotype had (p=0.044) (**Table 13**). Therefore, if the dose of MTX is higher than 2.5 g/m^2^, in patients with NK/T cell lymphoma and ANKL, it is necessary to detect the genotype of MTHFR rs1801131 and adjust the dose of MTX or lengthen the duration of leucovorin rescue in patients with the AA genotype, in order to decrease the risk of developing hematopoietic toxicity.

In conclusion, we identified an important influence of the SNPs of ABCB1 and MTHFR on MTX-related hematopoietic toxicity in adults with hematological malignancies. In order to optimize HD-MTX therapy and reduce related hematopoietic toxicity, it is necessary to detect the SNPs of MTHFR and ABCB1 before initiating HD-MTX and decide the optimal dose of MTX and duration of leucovorin rescue, according to the results of genetic tests and disease type in adults with hematological malignancies.

## Data Availability Statement

The original contributions presented in the study are included in the article/**Supplementary Material**. Further inquiries can be directed to the corresponding authors.

## Ethics Statement

The studies involving human participants were reviewed and approved by the medical ethics committees of Tongji Hospital. The patients/participants provided their written informed consent to participate in this study.

## Author Contributions

Z-YH, Z-QH, and LM designed the study. JH, LL, and JZ performed the data collection. LL performed the genotyping of SNPs. JH and HG performed the statistical analysis. JH wrote the original draft. Z-YH and Z-QH was responsible for the revision of the manuscript. All authors read and approved the manuscript.

## Funding

This study was supported by grants from the National Natural Science Foundation of China (grant number 81873430 to Z-YH, grant number 81772788 to Z-QH, and grant number 81974414 to Z-QH).

## Conflict of Interest

The authors declare that the research was conducted in the absence of any commercial or financial relationships that could be construed as a potential conflict of interest.

## Publisher’s Note

All claims expressed in this article are solely those of the authors and do not necessarily represent those of their affiliated organizations, or those of the publisher, the editors and the reviewers. Any product that may be evaluated in this article, or claim that may be made by its manufacturer, is not guaranteed or endorsed by the publisher.
